# Clinical utility of ^18^F-FDG PET/CT in the follow-up of a large cohort of patients with high-risk differentiated thyroid carcinoma

**DOI:** 10.1590/2359-3997000000285

**Published:** 2017-09-04

**Authors:** Ji H. Yang, Rui M. B. Maciel, Claudia C. D. Nakabashi, Carolina C. P. S. Janovsky, Rosalia P. Padovani, Danielle Macellaro, Cléber P. Camacho, Akemi Osawa, Jairo Wagner, Rosa Paula M. Biscolla

**Affiliations:** 1 Divisão de Endocrinologia Departamento de Medicina, Escola Paulista de Medicina Universidade Federal de São Paulo São Paulo SP Brazil Centro de Doenças da Tireoide e Laboratório de Endocrinologia Molecular e Translacional, Divisão de Endocrinologia, Departamento de Medicina, Escola Paulista de Medicina, Universidade Federal de São Paulo (EPM-Unifesp), São Paulo, SP, Brazil; 2 Departamento de Imagem Hospital Israelita Albert Einstein São Paulo SP Brazil Departamento de Imagem, Hospital Israelita Albert Einstein (HIAE), São Paulo, SP, Brazil

**Keywords:** Differentiated thyroid carcinoma, ^18^F-FDG PET/CT, radioiodine (RAI), whole-body scan (WBS), thyroglobulin (Tg)

## Abstract

**Objective:**

To evaluate the clinical utility of ^18^F-FDG PET/CT in patients with high-risk DTC.

**Subjects and methods:**

Single-center retrospective study with 74 patients with high-risk differentiated thyroid cancer (DTC), classified in 4 groups. Group 1: patients with positive sTg or TgAb, subdivided in Group 1A: negative RxWBS and no foci of metastases identified at conventional image (n = 9); Group 1B: RxWBS not compatible with suspicious foci at conventional image or not proportional to sTg level (n = 13); Group 2: patients with histological findings of aggressive DTC variants (n = 21) and Group 3: patients with positive RxWBS (n = 31).

**Results:**

^18^F-FDG PET/CT identified undifferentiated lesions and helped restage the disease in groups 1B and 2. The scan helped guide clinical judgment in 9/13 (69%) patients of group 1B, 10/21 (48%) patients of group 2 and 2/31 (6%) patients of group 3. There was no clinical benefit associated with group 1A. ^18^F-FDG PET/CT was associated with progressive disease.

**Conclusion:**

^18^F-FDG PET/CT is a useful tool in the follow-up of patients with high-risk DTC, mainly in the group of RxWBS not compatible with suspicious foci at conventional image or not proportional to sTg level and in those with aggressive DTC variants. Additionally, this study showed that ^18^F-FDG PET/CT was associated with progression and helped display undifferentiated lesions guiding clinical assessments regarding surgeries or expectant treatments.

## INTRODUCTION

The routine follow-up of patients with differentiated thyroid cancer (DTC) after surgery and radioiodine (RAI) remnant ablation comprises the measurement of serum thyroglobulin (sTg), cervical ultrasound (US), whole-body scan with ^131^I (WBS) or conventional imaging techniques, such as computed tomography (CT) or magnetic resonance imaging (MRI).

However, serum Tg levels may not be a reliable tool in some patients, mainly due to the presence of anti-thyroglobulin antibodies (TgAb) (1) or to undifferentiated tumors that do not secrete Tg. Similarly, WBS may fail to localize residual thyroid tissue in less differentiated tumors due to its impaired ability to concentrate RAI (2).

In recent years, combined ^18^F-fluorodeoxyglucose positron emission tomography/computed tomography (^18^F-FDG PET/CT) has emerged as a valuable tool in the follow-up of thyroid cancers. By correlating the metabolic information of ^18^F-FDG PET with the morphologic resolution of CT and due to the enhanced glucose metabolism in thyroid cancers, particularly in less differentiated tumors, this imaging technique has been employed beyond the classical indication of DTC patients with positive sTg and negative WBS. Furthermore, current applications extend to disease extension, including the detection of undifferentiated metastases (3), guidance of therapy assessments and prediction of prognosis (4-6). Some studies have shown that ^18^F-FDG PET and ^18^F-FDG PET/CT can induce changes in clinical management plans in 10-78% of patients with DTC (7-12), thereby improving clinical judgment. In the Brazilian population, there has been only one study using ^18^F-FDG PET/CT in thyroid cancer patients with negative WBS and positive sTg in a small patient sample (13).

The potential to induce changes in the clinical management and the lack of other studies in our country motivated this work. Therefore, the aim was to evaluate the clinical utility of the ^18^F-FDG PET/CT in a large cohort of patients with DTC in various groups.

## SUBJECTS AND METHODS

A total of 644 patients with DTC were referred, evaluated, treated and followed by a single team of physicians at the associated Thyroid Disease Centers in the Division of Endocrinology, Department of Medicine, *Escola Paulista de Medicina*, *Universidade Federal de São Paulo* and the *Instituto Israelita de Ensino e Pesquisa Albert Einstein* (in São Paulo, Brazil). In this population, 80 patients were submitted to ^18^F-FDG PET/CT scans from February 2008 to June 2013. Six patients were lost to follow-up; the medical records of the remaining 74 patients (who performed 95 total ^18^F-FDG PET/CT scans) were analyzed retrospectively. This study was approved by the Institutional Ethics Committee.

**Figure 1:**
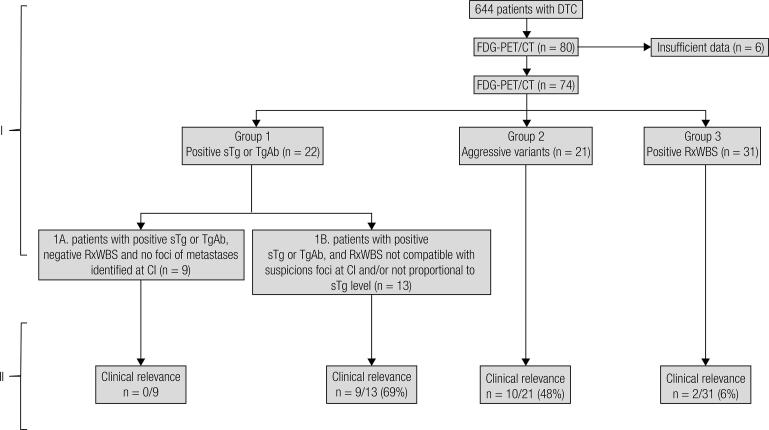
Schematic representation of the groups (I) and the clinical relevance (II).

According to the medical indications of ^18^F-FDG PET/CT, the 74 patients were classified in 4 groups (Figure 1, part I); clinical and epidemiological information is shown in Table 1:

**Table 1:** Clinical and epidemiological data

Clinical variables	Results
Sex	Female n = 57/Male n = 17
Total thyroidectomy	Yes n = 74
Lymph node resection	Yes: 57/No: 17
Age at diagnosis (years)	Median 40.9 (12-82)
Age at PET/CT (years)	Median 47.6 (16-85)
Follow up after PET/CT (months)*	Median 32.4 (7-72)
**Group 1A (n = 9)**	
sTg (ng/mL)	n = 6: 0.2-4.9 (Tg/LT4)/3.9-7.4 (Tg/TSH)
TgAb+	n = 2
Median of accumulated activity (mCi)	350 (200-750)
**Group 1B (n = 13)**	
sTg (ng/mL)	n = 7: 0,6-91 (Tg/LT4)/2.4-292 (Tg/TSH)
TgAb+	n = 5
Median of accumulated activity (mCi)	375 (230-700)
**Group 2 (n = 21)**	
sTg (ng/mL)	n = 20: 0.1-898 (Tg/LT4)/0.1-1000 (Tg/TSH)
TgAb+	n = 1
Median of accumulated activity (mCi)	250 (100-1000)
**Group 3 (n = 31)**	
sTg (ng/mL)	n = 30: 0.1-47 (Tg/LT4)/0.2-168 (Tg/TSH)
TgAb+	1
Median of accumulated activity (mCi)	450 mCi (150-800)

* From the first FDG-PET/CT; sTg: serum thyroglobulin; Tg/LT4: unstimulated Tg; Tg/TSH: stimulated Tg.

- *Group 1*. Patients with positive sTg or TgAb were subdivided in Group 1A (n = 9): negative post-therapeutic -131 whole body scan (RxWBS) and no foci of metastases identified at conventional image, and Group 1B (n = 13): RxWBS not compatible with suspicious foci at conventional image or not proportional to sTg level.
- *Group 2.* Patients with histological findings of aggressive DTC variants (n = 21): oncocytic (n = 3), poorly differentiated areas (n = 2), tall-cell (n = 4), diffuse sclerosing (n = 4), insular (n = 5) and solid variant (n = 3) with incomplete biochemical or structural disease.
- *Group 3*. Patients with positive RxWBS (n = 31): in this group, ^18^F-FDG PET/CT was performed to detect additional foci of undifferentiated metastases.

In the beginning of the study, 59 of 95 ^18^F-FDG PET/CT scans were performed after TSH stimulation (Tg/TSH): hypothyroidism, TSH > 30 mcUI/mL or after recombinant human TSH, rhTSH, Genzyme Transgenics Corp., Cambridge, Massachusetts. Over the course of follow-up, the literature demonstrated that despite studies showing that the number of positive scans and standard uptake value (SUV) increase under rhTSH stimulation, there was no conclusive evidence that those findings improve clinical management (12,14). Consequently, the remaining 36 scans were performed using LT4 (Tg/LT4). ^18^F-FDG PET/CT imaging and analysis were performed in accordance with the protocol described by Yamaga and cols. (13).

For the predictive value analysis, the following criteria defined by Hooft and cols. (15) were used: 1) histology/cytology; 2) US-FNAC for cervical lesions; 3) focal ^131^I-uptake; 4) pathognomonic bone scan or MRI for bone metastases; 5) CT/MRI for brain metastases; and 6) progression of radiological documented lesions suspect for malignancy. The results were considered positive in the presence of ^18^F-FDG uptake in suspected lesions (visualized on conventional image) or in those patients with biochemical disease. The results were considered negative if there was no ^18^F-FDG uptake. All patients underwent cervical US as the routine serial assessment, and suspicious cervical lesions were submitted to US-guided fine-needle aspiration cytology (US-FNAC) (16). Conventional imaging was performed during the follow-up if necessary (high levels of sTg measurements, WBS uptake, lung, retropharyngeal or bone suspicious metastases).

Serum Tg levels were measured by a highly sensitive chemiluminescence assay (Tg Access immunoassay, Beckman Coulter, Brea, CA) with a functional sensitivity of 0.1 ng/mL. TSH levels were measured using a third-generation assay that provided a functional sensitivity of 0.05 mUI/mL (17).

According to the combined and serial data of conventional imaging, the patient’s clinical status was classified as stable or progressive disease. Progressive disease was defined as an increase in tumor size during the follow-up, and stable disease was defined as stability of the lesions. Then, we analyzed the association between ^18^F-FDG uptake and progressive disease. We also studied the association between the PET/CT results and sTg levels using the cutoff recommended by ATA, 10 ng/mL.

For the continuous variables, the difference between positive and negative ^18^F-FDG PET/CT groups was assessed using the Mann-Whitney test. The ROC curve was used for the continuous variables to calculate the best cut-off point. The chi-square test was used to determine the differences in the frequency of the categorical variables. A *p* < 0.05 result was considered significant.

## RESULTS

### 18F-FDG PET/CT results and clinical relevance

**Group 1A: patients with positive sTg or TgAb, negative RxWBS and no foci of metastases identified at conventional image (n = 9)**

Ten scans were performed in this group. Although ^18^F-FDG PET/CT displayed 6 cervical positive lesions in only 3 patients, none was confirmed as metastasis based on US-FNAC. The sTg levels in this group were 0.2-4.9 ng/mL (Tg/LT4) and 3.9-7.4 ng/mL (Tg/TSH).

In conclusion, ^18^F-FDG PET/CT did not provide additional information in this group of patients.

**Group 1B: patients with positive sTg or TgAb and RxWBS not compatible with suspicious foci at conventional image or not proportional to sTg level (n = 13)**

Twenty ^18^F-FDG PET/CT scans were performed in this group of 13 patients, and nine of 13 presented positive ^18^F-FDG PET/CT scans (Table 2, patients 1-9). In those patients, the results helped clarify lesions visualized in conventional images that were RAI negative; in three of them, a second ^18^F-FDG PET/CT demonstrated the efficacy of the surgery indicated after the first scan results (Table 2, patients 1-3); in other three patients, the second ^18^F-FDG PET/CT was useful to show progression of the metastases (Table 2, patients 4, 6-7). The ^18^F-FDG PET/CT displayed, in all, cervical, mediastinal or retropharyngeal uptake in 4 patients who had confirmed metastatic lesions based on histological results (Table 2, patients 1-4). One patient (Table 2, patient 4) and the remaining 5 (Table 2, patient 5-9) presented diffuse pulmonary ^18^F-FDG uptake.

**Table 2:** Clinical and imaging characteristics of group 1B

N^**o**^	RxWBS uptake	PET	Tg/LT4	Tg/TSH	CT/RM findings	PET-CT findings	Management	Clinical Relevance
1	Negative	A	21	26	Thyroid bed Mediastinum Retropharingeaum	Thyroid bed Mediastinum Retropharingeaum	Surgery	Yes
		B	8.7	-	Thyroid bed Retropharingeaum	Thyroid bed Retropharingeaum	Expectant	
2	Negative	A	1.9	97	-	Mediastinum	Surgery	Yes
		B	3.8	-	-	Cervical	Expectant	
3	Negative	A	0.5*	-	-	Cervical	Surgery	Yes
		B	0.1*	-	Retropharingeaum	Retropharingeaum	Expectant	
4	Cervical	A	1.5	48	Thyroid bed Lung	Thyroid bed	Expectant	Yes
		B	14	173	Thyroid bed Mediastinum Lung	Thyroid bed Mediastinum Lung	Surgery	
5	Cervical	A	0.5*	-	Paratracheal Lung Esophagus	Paratracheal Lung Esophagus	Expectant	Yes
6	Negative	A	0.4*	-	Lung	Lung	Expectant	Yes
		B	0.1*	-	Lung	Progression of the lesions**	Expectant	
7	Negative	A	13	292	Lung	Lung	Expectant	Yes
		B	91	-	Lung	Progression of the lesions**	Expectant	
8	Negative	A	5000*	1000*	Hilar mass lung	Lung atelectasis	Expectant	Yes
9	Cervical	A	0.8*		Mediastinum Paratracheal Lunge	Mediastinum Paratracheal Lunge	Expectant	Yes
10	Cervical	A	0.6	4.8	Mediastinum Lung	Mediastinum Lung	Expectant	No
11	Negative	A	0.8	2.4	Mediastinum	None	Expectant	No
12	Negative	A	3.7	5.1	Lung	None	Expectant	No
13	Cervical	A	18	-	Lung	Lung	Expectant	No

**PET A, B:** 1^st^, 2^nd^ scans, respectively; **RxWBS:** Post-Therapeutic -131 Whole Body Scan; **Tg/LT4:** unstimulated Tg (ng/mL);

**Tg/TSH:** stimulated Tg (ng/mL). * TgAb positive; ** In relation to 1^st^ PET/CT.

In group 1B, we considered that the ^18^F-FDG PET/CT results helped localize metastases in 9/13 (69%).

**Group 2: patients with aggressive variants at the histological findings with incomplete biochemical or structural disease (n = 21)**

Twenty-seven scans were performed in this group of 21 patients. In 10 of 21 patients, ^18^F-FDG PET/CT provided relevant information.

*Oncocytic variant (n = 2):* The scan was indicated for an undetectable sTg and positive RxWBS cervical metastasis uptake, and there was ^18^F-FDG uptake in the cervical subcutaneous tissue. The other patient presented with RAI negative but suspicious pulmonary lesions on conventional images, and the ^18^F-FDG PET/CT scan showed lung uptake. Both lesions were confirmed as metastases based on histological analysis (Table 3, patients 1-2), indicating that ^18^F-FDG PET/CT provided relevant information.

**Table 3:** Clinical and imaging characteristics of group 2

N^**o**^	Histology	RxWBS uptake	PET	Tg/LT4	Tg/TSH	CT/RM findings	PET-CT findings	Management	Clinical Relevance
1	Oncocytic	Cervical	A	0.6	5.4	-	Subcutaneous	Surgery	Yes
2	Oncocytic	Cervical	A	278	-	Lung	Lung	Surgery	Yes
3	Oncocytic	Scapula	A	0.1	0.6	Scapula	Scapula	RAI	No
4	Poorly differentiated	Spine	A	14	767	-	Vertebra L4	RAI, RT	Yes
5	Poorly differentiated	Cervical	A	0.1	-	Retropharingeaum	None	Expectant	No
6	Insular	Ø RAI	A	511	1000	Lung	None	RAI	Yes
		Lung	B	44	-	Lung	None	RAI	
7	Insular	Ø RAI	A	3.3	18	Lung	None	RAI	Yes
8	Tall cell	Cervical Mediastinum Focal lung	A	13	95	Lung	Lung	Expectant	Yes
			B	134		Lung	Lung	Surgery	
			C	898	-	-	Brain	RT	
			D	379	-		Kidney, L2	Surgery	
9	Insular	Cervical Focal lung	A	1*	3*	Mediastinum Lung	Cervical Mediastinum Lung	Surgery TKI	Yes
			B	4*	6*	Mediastinum Lung	Progression of the lesions**	Expectant	
10	Tall cell	Negative	A	89	-	Lung	Lung	Surgery	Yes
			B	104	635	Lung	Lung	Expectant	
11	Tall cell	Cervical	A	17	42	Lung	Lung	Expectant	Yes
12	Solid trabecular	Cervical	A	44	66	Lung	Lung	Expectant	Yes
13	Solid trabecular	Negative	A	0.2	0.2	-	None	Expectant	No
14	Solid trabecular	Mediastinum	A	0.2	-	Mediastinum	Cervical	Expectant	No
15	Diffuse sclerosing	Lung	A	0.1	2.7	-	Thyroid bed	Expectant	No
16	Diffuse sclerosing	Negative	A	1.9	271	Paratracheal	None	PEI	No
17	Diffuse sclerosing	Cervical	A	0.2	0.1	-	None	Expectant	No
18	Diffuse sclerosing	Negative	A	1.9	4.3	-	None	Expectant	No
19	Insular	Cervical	A	428	-	Lung	None	Expectant	No
20	Insular	Ø RAI	A	65	-		Cervical	Expectant	No
21	Tall cell	Cervical	A	2.4	-	-	Cervical	Expectant	No

**PET A, B, C, D:** 1^st^, 2^nd^, 3^rd^ and 4^th^ scans, respectively; **PEI:** percutaneous injection of ethanol; **RAI:** radioiodine; **RxWBS:** Post-Therapeutic -131 Whole Body Scan; Ø **RAI:** without radioiodine; **RT:** radiotherapy; Tg/LT4: unstimulated Tg (ng/mL); **Tg/TSH:** stimulated Tg (ng/mL); **TKI:** tyrosine-kinase inhibitor. * TgAb positive; ** In relation to 1st PET/CT.

*Poorly differentiated areas on histology (n = 1):* the patient presented a positive RxWBS lumbar vertebrae metastasis with accelerated elevation of sTg, and the positive ^18^F-FDG uptake suggested possible tumor undifferentiation. This patient also presented with cerebral metastasis and had been treated with cerebral and vertebrae radiotherapy (Table 3, patient 4).

**Figure 2:**
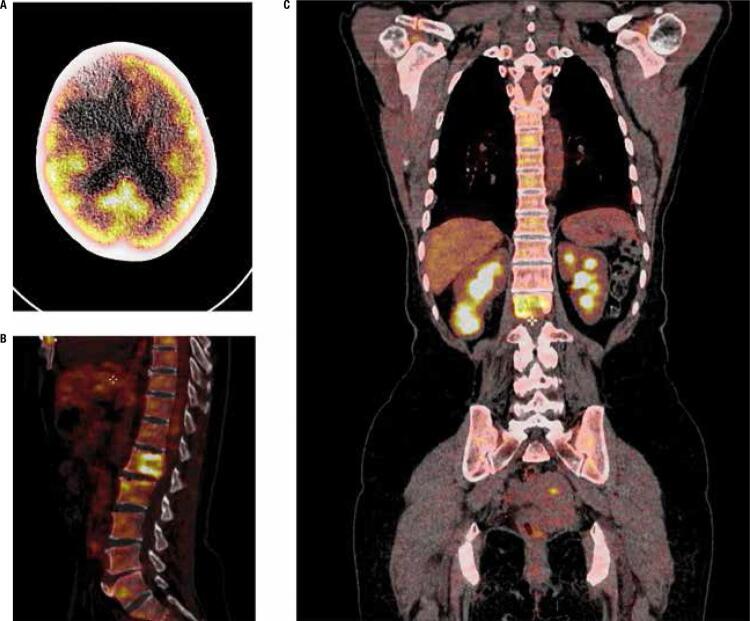
18F-FDG PET/CT images show increased metabolic activity in 62-y-woman: A. Expansive lesion on frontal lobe of the brain; B. Lytic lesion on L2 vertebral body; C. Right lower renal mass. (Table 3, patient 8).

*Other aggressive variants (n = 7):* ^18^F-FDG PET/CT provided relevant information in 7 patients. Two patients without previous RAI treatment underwent paired ^18^F-FDG PET/CT and DxWBS scans. A ^18^F-FDG negative scan combined with positive RAI uptake endorsed the first RAI treatment indication (Table 3, patients 6-7). The other 5 patients had ^18^F-FDG uptake in pulmonary nodules, suggesting undifferentiated metastases (Table 3, patients 8-12). Three patients underwent resection of the ^18^F-FDG positive metastases (Table 3, patients 8-10), but despite treatment, the disease progressed in all patients, and one patient presented with renal metastasis confirmed on histopathology, cerebral metastasis refractory to radiotherapy and death (Table 3, patient 8, Figure 2). In this group, the ^18^F-FDG PET/CT results contributed to the clinical management in 10/21 patients (48%).

**Group 3: patients with positive RxWBS (n = 31)**

This group included 31 patients with positive WBS who underwent 39 scans. There was no additional ^18^F-FDG uptake suggestive of metastases in respect to RxWBS or conventional images. However, 2 patients with previous RxWBS positive pulmonary disease and increasing sTg/TgAb levels showed pulmonary ^18^F-FDG uptake, suggesting tumor undifferentiation. In this group of 31 patients, the ^18^F-FDG PET/CT results delineated prognosis in 2 patients (6%).

The clinical relevance of each group is summarized in Figure 1, part II.

### 18F-FDG PET/CT result as predictor of progressive disease

From the 95 ^18^F-FDG PET/CT scans, we found 61 positive, 28 negative and 6 indeterminate scans. All eighteen patients who presented with progressive disease showed ^18^F-FDG uptake (100%) compared to twenty-nine of fifty-six patients who were stable (52%). The ^18^F-FDG uptake was associated with progressive disease (*p* = 0.0004).

### 18F-FDG PET/CT results and sTg measurements

The median serum Tg/LT4 was 9.4 ng/mL (0.1-898) in the positive PET/CT patients, compared to 0.8 ng/mL (0.1-44) in the PET/CT negative group (*p* = 0.001). In analyzing the Tg/TSH level, the median level in the positive PET/CT patients was 26 ng/mL (0.9-1,000), in contrast to 5.1 ng/mL (0.1-271) in the PET/CT negative group (*p* = 0.003) (Table 4A). In analyzing each group separately, there was a significant difference between the positive and negative PET/CT results in group 1 (for both Tg/LT4 and Tg/TSH) and group 2 (only for Tg/LT4). If we used the sTg level of 10 ng/mL, there were abnormal PET/CT results in 52% of all patients with Tg/LT4 levels ≤ 10 ng/mL and in 90% of patients with Tg/LT4 levels of > 10 ng/mL (p < 0.001) (Table 4B). For Tg/TSH, the outcome was 38% in Tg/TSH levels of ≤ 10 ng/mL versus 74% if Tg/TSH was > 10 ng/mL (p < 0.009). For each group, separately, there was a significant difference in only group 1 (for both Tg/LT4 and Tg/TSH) and group 2 (only for Tg/LT4).

**Table 4:** – A. The median of thyroglobulin level from 18F-FDG PET/CT positive (FDG +) and negative (FDG -) patients. B. 18F-FDG PET/CT positive result according to the cutoff of sTg = 10 ng/mL.

A									
		FDG +	FDG -		p
All patients	Tg/LT4 (ng/mL)	9.4 (0.1-898)	0.8 (0.1–44)		< 0.01
	Tg/TSH (ng/mL)	26 (0.9-1000)	5.1 (0.1–271)		< 0.01
Group 1	Tg/LT4 (ng/mL)	6.9 (0.5-91)	1.4 (0.1-4.9)		< 0.05
	Tg/TSH (ng/mL)	72.5 (3.9-292)	5.1 (2.4-7.4)		< 0.05
Group 2	Tg/LT4 (ng/mL)	59.1 (0.2-898)	0.2 (0.1-44)		< 0.05
	Tg/TSH (ng/mL)	95 (0.4-1000)	0.6 (0.1-271)		> 0.05
Group 3	Tg/LT4 (ng/mL)	2.2 (0.1-47)	1.0 (0.1-7.9)		> 0.05
	Tg/TSH (ng/mL)	13.9 (1.1-168)	8.0 (0.7-38)		> 0.05

Tg/LT4: unstimulated Tg; Tg/TSH: stimulated Tg.									
**B**					

	**^**18**^ F-FDG PET/CT positive result**				

	**Tg ≤ 10 ng/mL**		**Tg > 10 ng/mL**		**p**

**All patients**	Tg/LT4	27/52 (52%)	Tg/LT4	21/22 (95%)	< 0.01
	Tg/TSH	10/26 (38%)	Tg/TSH	20/27 (74%)	< 0.01
**Group 1**	Tg/LT4	6/13 (46%)	Tg/LT4	5/5 (100%)	< 0.05
	Tg/TSH	1/8 (13%)	Tg/TSH	5/5 (100%)	< 0.01
**Group 2**	Tg/LT4	5/11 (45%)	Tg/LT4	13/14 (93%)	< 0.01
	Tg/TSH	4/7 (57%)	Tg/TSH	7/9 (78%)	> 0.05
**Group 3**	Tg/LT4	17/29 (59%)	Tg/LT4	3/3 (100%)	> 0.05
	Tg/TSH	5/11 (45%)	Tg/TSH	8/13 (62%)	> 0.05

Tg/LT4: unstimulated Tg; Tg/TSH: stimulated Tg.					

## DISCUSSION

High metabolic activity revealed by ^18^F-FDG avidity represents advanced tumor and undifferentiation. In these cases, poorly differentiated follicular cells might lose the ability to concentrate RAI, synthesize sTg, and progressively enhance glucose metabolism due to high cell activity and metabolic demand. In this way, ^18^F-FDG PET/CT has become a powerful tool to improve staging and tumor aggressiveness and investigate undifferentiated lesions that do not take up radioiodine, denoting important diagnostic and prognostic implications (18-20).

The classical indication to perform ^18^F-FDG PET/CT in thyroid cancer patients is positive sTg measurements with negative WBS uptake (6). In the literature, ^18^F-FDG PET/CT provides additional information not revealed by traditional images in 21-71% of patients, mostly in negative WBS (7,8), and in 13-50% of patients with positive TgAb (21,22).

In our study, we analyzed the classical indication of ^18^F-FDG PET/CT in 22 patients with positive sTg or TgAb, negative RxWBS and no foci of metastases identified at conventional image (Group 1A, n = 9) and those with positive sTg or TgAb and RxWBS not compatible with suspicious foci at conventional image or not proportional to sTg level (Group 1B, n = 13). In group 1A, ^18^F-FDG PET/CT did not detect additional metastases. The low sTg levels (0.2-4.9 ng/mL), even under stimulation (3.9-7.4 ng/mL), combined with microscopic metastasis might explain the lack of ^18^F-FDG-avid lesions, as ^18^F-FDG is limited in detecting minimal disease (under 1.0 cm). However, the ^18^F-FDG PET/CT scan helped unveil undifferentiated cervical, lung and mediastinal metastases in 9 patients (69%) in group 1B. The positive FDG uptake observed in this subgroup was associated with higher levels of sTg (Tg/LT4: 0.6-91 ng/mL and Tg/TSH: 2.4-292 ng/mL) and higher dimensions of metastasis.

In the subgroup 1B, surgery was possible in 4 of 9 patients with positive ^18^F-FDG PET/CT scan results. As described by Hall and Kloos (3), the ideal of ^18^F-FDG, to identify resectable lesions to pursue a cure, should be attempted as undifferentiated lesions are less likely to respond to RAI, and additional surgery can lead to a higher rate of full remission during follow-up (23). The other 6 patients presented diffuse pulmonary ^18^F-FDG uptake, and there was no role for surgery.

The other aspect to consider is the behavior of aggressive histological variants. Those variants have unfavorable prognosis as they feature low iodine avidity and aggressive clinical behavior with more local and distance recurrences, less disease-free intervals and shorter survivals, requiring close follow-up and continued surveillance to pursue occult metastases. Publications regarding ^18^F-FDG PET/CT and aggressive variants describe these subtypes as more ^18^F-FDG-avid than RAI tumors. Concerning oncocytic cell tumors, 80% of patients have no iodine-avid tumor (24), and therefore, ^18^F-FDG PET/CT is a valuable tool for screening occult recurrence, evaluating prognosis, and providing additional images not presented by WBS or conventional image (25,26). In regard to the other aggressive subtypes, few studies consider ^18^F-FDG PET/CT as a useful guide in the management of insular (27), sclerosing diffuse (28) and tall cell (29) variants. Treglia and cols. (30) concluded that the ^18^F-FDG PET scan usefulness is clear for the oncocytic cell*,* uncertain for poorly differentiated cancers and suggestive in the other aggressive forms. The concept of tumor undifferentiation was also observed in patients in group 2 (n = 21). In our study, the results corroborate the findings in the literature. The higher FDG uptake presented in this group can be attributed to more undifferentiated thyroid tumors with more avid uptake for ^18^F-FDG and high levels of sTg. Pryma and cols. (25) suggested that ^18^F-FDG PET/CT could be indicated in oncocytic cell carcinoma in postoperative staging and as follow-up in patients with an increase in sTg or recurrent disease, whereas Nascimento and cols. (29) recommended routine early postoperative ^18^F-FDG PET/CT concomitantly with RxWBS in all patients with aggressive histological DTC.

^18^F-FDG and RAI may function as complementary tools in DTC (9,31) to investigate additional undifferentiated metastases. However, in our cohort, we did not find additional metastases visualized by WBS. WBS-positive patients have no classical indication for ^18^F-FDG PET/CT and cost-efficacy must be considered in WBS positive group patients.

ATA recommends ^18^F-FDG PET/CT in high-risk DTC patients with elevated sTg, generally Tg/TSH > 10 ng/mL (6). If Tg/TSH is ≤ 10 ng/mL, the sensitivity of PET/CT is low, ranging from less than 10% to 30% (6). In our data, 38% of all scans performed with Tg/TSH of ≤ 10 ng/mL and 13% of group 1 were positive, similar to the literature data. In contrast, 74% of PET/CT performed with Tg/TSH of > 10 ng/mL provided positive results (as was the case for 100% of group 1). Regarding unstimulated Tg analysis, Tg/LT4 > 10 ng/mL was associated with higher lesion detection in overall patients and groups 1 and 2 when compared to Tg/LT4 ≤ 10 ng/mL. As a matter of fact, more important than the influence of rhTSH or thyroid hormone withdrawal in ^18^F-FDG PET/CT is the presence of high levels of Tg (Tg/TSH or Tg/LT4 > 10 ng/mL). Over the last years, it has been demonstrated that both strategies, with or without TSH stimulation, do not considerably lead to management changes (12,32).

Additional factors than sTg that influence ^18^F-FDG PET/CT sensitivity are tumor de-differentiation and larger tumor burden (6), as we have seen in our results. Additionally, the scanning is limited in detecting minimal disease (generally less than 1 cm) and well-differentiated metastases, resulting in false negative outcomes. It is well known that inflammatory lesions can take up FDG, and there may be false positive results. All these features should be considered with care to avoid misjudgments. The frequency of false positive lesions in the literature varies among studies from 0 to 39% (6), and this high number justifies the PET/CT results through the combined data of clinical, laboratorial, conventional image and cytological/histological information to guide ongoing clinical assessments.

The present work has some limitations. First, this was a retrospective study, and direct comparison of the detection rate of metastases between ^18^F-FDG PET/CT and other diagnostic methods was not the design of this study. Additionally, we had no cytological or pathological confirmation of all lesions with ^18^F-FDG uptake. Based on the serial evaluation of thyroid cancer patients with laboratorial and image exams to assess tumor growth, invasive procedure to confirm the metastases is seldom necessary.

In conclusion, ^18^F-FDG PET/CT results changed the management in 28% (21/74) of patients, mostly in 1B group, patients with RxWBS not compatible with suspicious foci at conventional image or not proportional to sTg level (69%, 9/13) and in group 2, patients with aggressive histological variant patients (48%, 10/21), confirming the literature indications that ^18^F-FDG PET/CT is more useful in these two groups of patients. For group 1A patients, with positive sTg or TgAb, negative RxWBS and no foci of metastases identified at the conventional image, ^18^F-FDG PET/CT was not useful, probably due to low levels of sTg and low tumor burden. Additionally, this study showed that ^18^F-FDG uptake was associated with progressive disease and helped display undifferentiated lesions guiding clinical assessments with respect to surgeries or expectant treatments.
